# Catalytic Conversion of Xylo-Oligomers to Furfural in Pulping Pre-Hydrolysis Liquor Using a Hydroxyl-Functionalized Covalent Organic Framework

**DOI:** 10.3390/polym17081102

**Published:** 2025-04-18

**Authors:** Kai Zhang, Huanmei Xia, Guangyao Cheng, Peng Gan, Yuan Ju, Baozhen Guo, Jingli Yang, Chengcheng Qiao, Jixiang Lin, Jiachuan Chen

**Affiliations:** 1 State Key Laboratory of Green Papermaking and Resource Recycling, Key Laboratory of Pulp and Paper Science and Technology of Education Ministry, Qilu University of Technology (Shandong Academy of Sciences), Jinan 250353, China; zhangkai2018@qlu.edu.cn (K.Z.); 19861904802@163.com (H.X.); jy19981208jy@163.com (Y.J.); gbz1985@163.com (B.G.); 15148161132@163.com (J.Y.); qiao_dy@126.com (C.Q.); chenjc@qlu.edu.cn (J.C.); 2 Key Laboratory of Clean Pulp & Papermaking and Pollution Control of Guangxi, College of Light Industrial and Food Engineering, Guangxi University, Nanning 530004, China; 3 Key Laboratory of Saline-Alkali Vegetation Ecology Restoration, Ministry of Education/College of Landscape Architecture, Northeast Forestry University, Harbin 150040, China

**Keywords:** pulp and paper industry, biomass refinery plant, functionalization, covalent organic frameworks, xylo-oligomers, furfural

## Abstract

With the rapid development of biorefinery technology, the efficient conversion of lignocellulose into high-value platform chemicals is of great significance for enhancing the value of renewable carbon resources. In this study, a hydroxyl-functionalized covalent organic framework (COF), TAPB-DHPA, was synthesized via an in situ method and innovatively applied to the catalytic conversion of xylo-oligosaccharides (XOS) into furfural. The results demonstrated that TAPB-DHPA possesses a large specific surface area, a well-developed porous structure, and excellent thermal stability, with abundant Brønsted acid (B acid) sites, exhibiting outstanding catalytic activity. Under optimal conditions, including a catalyst loading of 0.16 wt%, a reaction temperature of 180 °C, and a reaction time of 3 h, a furfural yield of up to 65.4% was achieved. The high selectivity was primarily attributed to the p-π conjugation effect between the benzene ring and the phenolic hydroxyl group, which enhanced the ionization ability of hydroxyl hydrogen, thereby effectively promoting the hydrolysis of XOS and subsequent dehydration. Furthermore, TAPB-DHPA exhibited excellent recyclability and stability, maintaining a furfural yield of over 59.9% after six cycles. This study provides new insights into the application of functionalized COF in biomass catalytic conversion and contributes to the green transformation of the pulp and paper industry into a biorefinery-based model.

## 1. Introduction

With the depletion of global non-renewable resources, the efficient utilization of abundant and renewable lignocellulosic biomass has become a research focus in the fields of energy and materials [1]. The pulp and paper industry has achieved effective separation of lignocellulosic components through clean pulping technologies. By integrating biomass refining techniques, it can not only produce traditional pulp and paper products but also develop high-value-added biomass chemicals or bio-based materials [2], accelerating the transformation and upgrading of the pulp and paper industry into a more intensive biomass refining industry.

The pre-hydrolysis kraft process, due to its high lignin removal efficiency, has become the preferred technology for producing dissolving pulp [3]. The core step, pre-hydrolysis, effectively removes a large portion of hemicellulose from the raw material, enhancing the penetration of chemical reagents and improving the quality of dissolving pulp [4,5]. However, the pre-hydrolysis process generates a large amount of pre-hydrolysis liquor, which is typically treated through combustion for heat recovery or discharged after treatment, leading to waste of valuable biomass resources, increased production costs, and potential water pollution.

Xylo-oligosaccharides (XOS), a key component of pre-hydrolysis liquor derived from hemicellulose degradation, have attracted significant attention for their conversion into high-value chemicals [6,7]. Furfural, a key platform chemical, has extensive application potential in bio-based chemicals, fuels, and pesticides [8,9]. The production of furfural can not only promote the development of the biomass economy but also facilitate the green transformation and industrial upgrading of the pulp and paper industry. Currently, industrial production of furfural primarily relies on traditional chemical catalysis, which suffers from low substrate conversion rates, high energy consumption, and severe environmental pollution [10]. Therefore, developing efficient and green catalysts for the conversion of biomass sugars into furfural, with the aim of increasing furfural yield and reducing energy consumption, has become the key to overcoming the current industrial production bottlenecks.

Covalent organic frameworks (COFs) have shown great promise in catalysis due to their unique structural features, such as high surface area, tunable porosity, excellent chemical stability, and rich functionalization potential. Through rational design and functional modification, COFs can integrate specific catalytic active sites, effectively promoting the conversion of biomass resources into high-value products. Peng et al. synthesized sulfonic acid-functionalized TFP-DABA-COF-SO_3_H, which achieved efficient conversion of fructose to 5-hydroxymethylfurfural (HMF) in a dimethyl sulfoxide (DMSO) solvent system, with a high yield of 97%. Its excellent catalytic performance is attributed to the Brønsted acidity provided by the sulfonic groups [11]. Du et al. innovatively proposed a one-pot self-assembly strategy to construct a BIL-COF composite catalytic system with ionic liquid loading characteristics by precisely anchoring Brønsted acidic ionic liquid [PSMIm][HSO_4_] within imine-linked COF frameworks [12]. Under optimized conditions, this system achieved an impressive 97% yield in the conversion of sorbitol to isosorbide. Despite significant progress in developing functionalized COFs for biomass conversion into high-value chemicals, their application in furfural production remains limited. To address this gap, our team employed molecular design principles to develop hydroxyl-functionalized TAPT-DHPA-COF using 2,5-dihydroxyterephthaldehyde containing B-acid sites as the functional monomer. This catalyst exhibited an exceptional 86.7% furfural yield in xylose dehydration reactions. We further developed TP-DAB-COF, enriched with phenolic hydroxyl and carboxylic acid groups, which maintained a furfural yield of 63.49% when applied to complex xylan substrates [13,14]. It is noteworthy that although functionalized COFs have demonstrated outstanding catalytic performance at the laboratory scale, their practical application in the conversion of industrial lignocellulosic waste remains unexplored. Systematic investigations on catalytic mechanisms, structure–activity relationships, and industrial feasibility are still required to fully unlock their potential in biomass valorization.

Therefore, this study focuses on the high-value utilization of XOS in pre-hydrolysis liquor, employing an in situ synthesis strategy with 1,3,5-tris(4-aminophenyl) benzene (TAPB) and 2,5-dihydroxyterephthalaldehyde (DHPA) as monomers to prepare hydroxyl-functionalized COFs for catalyzing the conversion of XOS into furfural. By optimizing the catalytic reaction conditions, the conversion rate of XOS and the yield of furfural will be improved. Comprehensive characterization of the catalysts’ structural and chemical properties will be conducted to explore the structure–activity relationship and elucidate the mechanism by which functionalized COFs efficiently catalyze the production of furfural from XOS. This study aims to achieve efficient and sustainable conversion of XOS from pulping pre-hydrolysis liquor into furfural, thereby providing a solid data foundation and theoretical basis for the green development of the pulp and paper industry and its transition toward integrated biorefinery systems.

## 2. Materials and Methods

### 2.1. Materials and Chemicals

The XOS used in this study was derived from the pre-hydrolysis liquor of eucalyptus wood dissolving pulp from a paper mill in Shandong Province. The chemical reagents used include 1,3,5-tris (4-aminophenyl) benzene (TAPB), 1,2-dichlorobenzene, n-butanol, glacial acetic acid, tetrahydrofuran (THF), 1,4-dioxane (DIO), toluene (TOL), γ-valerolactone (GVL), and furfural, purchased from Aladdin Chemistry (Shanghai, China). 2,5-dihydroxyterephthalaldehyde (DHPA) was purchased from Macklin (Shanghai, China).

### 2.2. Characterization of XOS

The moisture content of XOS was determined by the direct drying method. Specifically, 2 g of sample was placed in a crucible and dried in an oven at 105 °C for 4 h. The sample was then cooled in a desiccator for 0.5 h and weighed. This drying–cooling–weighing procedure was repeated by returning the sample to the 105 °C oven for an additional 1 h, followed by cooling and reweighing. The process was continued until the difference between successive weights was ≤2 mg. The final mass loss was recorded as the moisture content.

The purity of XOS was determined according to the method established by the National Renewable Energy Laboratory (NREL) (NREL/TP-510-42618, Golden, CO, USA). The specific procedure was as follows: 0.3 g of the sample was placed in a 10 mL crucible, followed by the addition of 3 mL of 72% sulfuric acid. After thorough swelling for 30 min, 84 mL of distilled water was added to dilute the solution to 4%. The mixture was then reacted at 121 °C for 1 h. The xylose content was measured using high-performance liquid chromatography (HPLC, LC-20AT, Shimadzu, Kyoto, Japan) equipped with an Aminex HPX-87H column, Agilent, CO, USA, and the purity of XOS was calculated accordingly. All experiments were conducted in triplicate, and the mean values were reported to minimize experimental variability. Fourier transform infrared spectroscopy (FT-IR, ALPHA II, Bruker, Billerica, MA, USA) was used to record the infrared absorption spectrum of XOS in the range of 500–4000 cm^−1^ to characterize its structural features. The crystalline structure of the raw material was analyzed using X-ray diffraction (XRD, Smartlab SE, Rigaku, Tokyo, Japan), with testing parameters set to a step size of 0.02°, a scanning speed of 20°/min, and on chromatography (GPC, LC-20AD, Shimadzu, Kyoto, Japan).

### 2.3. Preparation of Functionalized COF

A hydroxy-functionalized COF was synthesized via an in situ synthesis strategy using a solvothermal method. The specific procedure is as follows: 0.48 mmol of TAPB, 3 mL of n-butanol, 0.72 mmol of DHPA, and 3 mL of 1,2-dichlorobenzene were sequentially added to a Pyrex tube and sonicated for 20 min to ensure uniform dispersion of the reactants. Next, 0.6 mL of glacial acetic acid was added to facilitate the Schiff base reaction. The reaction system was degassed through three freeze–pump–thaw cycles. The Pyrex tube was then vacuum-sealed and placed in a 120 °C oven for 72 h. After the reaction, the Pyrex tube was removed and cooled to room temperature. The product was extracted continuously for 3 h using THF as the solvent in a Soxhlet extractor. After extraction, the precipitate was thoroughly washed with anhydrous ethanol. Finally, the precipitate was dried in a vacuum oven at 80 °C for 12 h to obtain the hydroxyl-functionalized COF, designated as TAPB-DHPA, with a yield of 91.2%.

### 2.4. Characterization of Functionalized COF

The crystalline structure of TAPB-DHPA was analyzed using XRD with a scanning range set from 2° to 50°. FT-IR was employed to characterize the characteristic chemical bonds of TAPB-DHPA, with a scanning wavelength range of 400–4000 cm^−1^. The thermal stability of TAPB-DHPA was evaluated by thermogravimetric analysis (TGA, Q50/DSAQ20, TA Instruments, New Castle, DE, USA) under nitrogen atmosphere, using a heating rate of 10 °C/min up to 800 °C. The microscopic morphology of TAPB-DHPA was observed using scanning electron microscopy (SEM, Regulus8220, Hitachi High-Tech, Tokyo, Japan). The acidity of TAPB-DHPA was determined by ammonia temperature-programmed desorption (NH_3_-TPD, Microtrac BelCata II, MicrotracBEL, Osaka, Japan). The nitrogen adsorption–desorption data obtained at 77.4 K using the Brunauer–Emmett–Teller (BET, Micromeritics ASAP 2460, Micromeritics Instrument, Norcross, GA, USA) method were used to calculate the specific surface area and pore size distribution.

### 2.5. Catalytic Conversion of XOS into Furfural

The catalytic conversion of XOS to furfural was carried out in a high-temperature reactor (Parr, 5500 Series, Parr Instrument, Moline, IL, USA). The experimental procedure was as follows: A total of 30 mL of a mixed solvent system composed of an organic solvent and water, along with 0.8 g of XOS, was added to the reactor and stirred to ensure complete dissolution. Subsequently, TAPB-DHPA was introduced, and the system was purged with nitrogen. The stirring speed was set to 500 rpm, and the catalytic reaction was conducted at the designated temperature. After the reaction, the mixture was filtered using a vacuum filtration device to separate the filtrate from the solid catalyst. The furfural content in the filtrate was analyzed using HPLC equipped with a UV detector and a refractive index detector. Specifically, the furfural yield was calculated on a dry ash-free basis of the XOS, with the moisture content and inorganic impurities in raw XOS subtracted to determine its theoretical conversion capacity. Furfural was quantified using an external standard calibration curve with HPLC, ensuring precise determination of product concentration. All experiments were conducted in triplicate, and the mean values were reported to minimize experimental variability. The solid catalyst was thoroughly washed with n-butanol and 1,2-Dichlorobenzene then dried in an oven for reuse in subsequent catalytic cycles.

## 3. Results and Discussion

### 3.1. Basic Properties of XOS

Table 1 presents the basic properties of the XOS samples, including moisture content, purity, molecular weight, and polydispersity index (PDI). The XOS extracted from the hardwood pulping pre-hydrolysis liquor has a moisture content of 0.44% and a purity of 96.7%. Its PDI value is 1.07, indicating a relatively narrow molecular weight distribution. Further analysis revealed that the XOS sample exhibited a weight-average molecular weight (Mw) of 306 g/mol and a number-average molecular weight (Mn) of 286 g/mol. These values are close to the molecular weight of xylobiose (282.24 g/mol), indicating that the XOS sample is likely primarily composed of xylobiose units [15].

**Figure 1:**
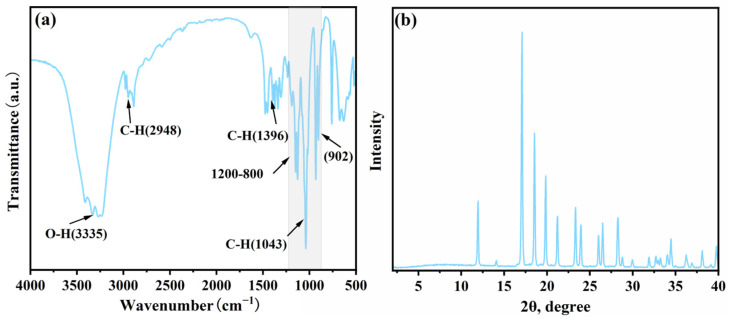
(**a**) FT-IR spectrum and (**b**) XRD pattern of XOS.

Figure 1a shows the FT-IR spectrum of XOS. The absorption peaks at 3335 cm^−1^ and 2948 cm^−1^ correspond to the stretching vibrations of hydroxyl (O-H) groups and methyl (CH_3_) or methylene (CH_2_) groups in polysaccharides, respectively. The absorption peak at 1396 cm^−1^ is associated with the bending vibration of C-H bonds in carbohydrate molecules [16,17]. Additionally, the absorption peaks in the range of 1200–800 cm^−1^ are considered to be in the “fingerprint” region of polysaccharides, mainly consisting of coupled vibrations of C-O-C, C-C-O, and C-OH [16,18]. The strong absorption peak observed at 1043 cm^−1^ is a characteristic peak of xylan. Finally, the absorption peak at 902 cm^−1^ represents the characteristic absorption of β-glycosidic bonds, further confirming that the sugar units in XOS are connected by β-glycosidic bonds [19].

As shown in Figure 1b, the XRD of XOS demonstrates excellent crystallinity, with no diffraction peaks observed for amorphous polymers. Typically, xylan components that are not substituted by side chains easily form hydrated or anhydrous crystals, while xylan molecules with side chain groups tend to inhibit crystallization, resulting in an amorphous structure [20]. Therefore, it can be concluded that the pentose molecules in XOS are almost unmodified by side chain groups, thus maintaining a high degree of crystallinity.

### 3.2. Structure and Properties of TAPB-DHPA

**Figure 2:**
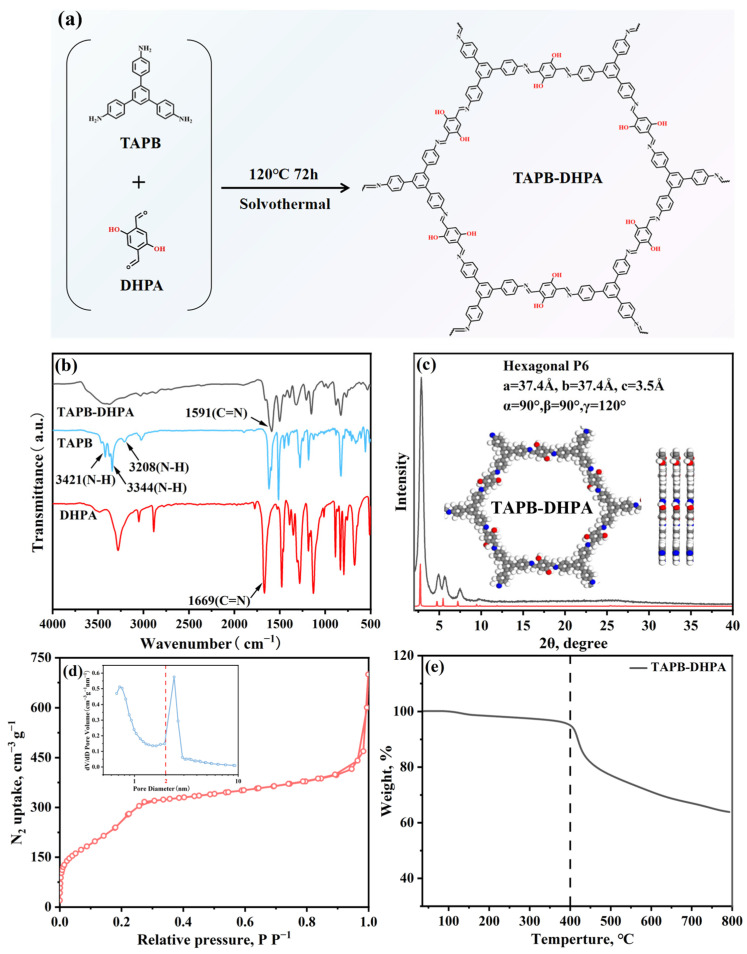
Synthesis and characterization of TAPB-DHPA: (**a**) solvothermal route; (**b**) FT-IR; (**c**) XRD; (**d**) N_2_ adsorption–desorption isotherms; (**e**) TGA.

As shown in Figure 2a, TAPB-DHPA was synthesized from monomers containing hydroxyl functional groups through a Schiff base reaction using an in situ synthesis strategy. To systematically characterize the structural properties and microscopic morphology of TAPB-DHPA, various techniques were employed, including FT-IR, XRD, BET, NH_3_-TPD, TGA, and SEM. Through these characterization methods, we were able to conduct an in-depth analysis of the chemical structure, crystalline structure, specific surface area, acidic properties, thermal stability, and surface morphology of the TAPB-DHPA material.

Figure 2b shows the FT-IR spectra of TAPB, DHPA, and the condensation product TAPB-DHPA. In the TAPB spectrum, the strong stretching vibration peaks at 3421, 3344, and 3208 cm^−1^ are attributed to N-H bond stretching vibrations. The stretching vibration peak at 1669 cm^−1^ in DHPA corresponds to the C=O group. The condensation of TAPB and DHPA to form TAPB-DHPA was accompanied by the complete disappearance of characteristic FTIR peaks corresponding to TAPB and DHPA with the appearance of a prominent C=N stretching vibration at 1591 cm^−1^, confirming quantitative formation of a Schiff base between aldehyde and amine groups [21]. This reaction in acetic acid follows a stepwise mechanism: (1) protonation of amine groups reduces the electron density of the lone pair while maintaining partial nucleophilicity; (2) hydrogen bonding between the aldehyde carbonyl oxygen and acetic acid polarizes the C=O bond, enhancing the electrophilicity of the carbonyl carbon; (3) nucleophilic attack by the amine lone pair on the carbonyl carbon generates a tetrahedral intermediate, which then undergoes acid-catalyzed proton transfer and dehydration to yield the final imine linkage.

The crystallinity of TAPB-DHPA was studied using XRD. As shown in Figure 2c, the material exhibits significant diffraction peaks at 2θ = 2.8°, 4.9°, 5.6°, 7.4°, and 25.5°, corresponding to the (100), (110), (200), (210), and (001) crystal planes, respectively, indicating that TAPB-DHPA possesses good crystallinity [22]. In imine-based COFs containing hydroxyl groups, the hydrogen bonds formed between phenolic hydroxyl groups (-OH) and imine bonds (C=N) significantly enhance crystallinity by promoting oriented molecular arrangement and strengthening interlayer coupling [23]. Specifically, the strong directionality of hydrogen bonds forces adjacent aromatic rings into an AA stacking mode with ordered alignment, thereby reducing the rotational freedom of the molecules. Concurrently, the periodic hydrogen bonding network enhances lattice energy through synergistic effects, suppresses lattice distortions, and decreases defect density. Furthermore, these hydrogen bonds act as pre-organizational templates during crystallization, accelerating nucleation and guiding anisotropic growth, which results in the formation of long-range ordered crystalline structures with high stability. The TAPB-DHPA framework, containing phenolic hydroxyl groups and imine bonds to form hydrogen bonds (OH···N=C), is identified as a key factor contributing to its robust crystallinity [24]. To further validate the crystallographic structure of TAPB-DHPA, its crystal structure was simulated using Materials Studio 2020 software, assuming its space group to be P6 and setting the corresponding unit cell parameters. The simulated XRD pattern closely matched the experimental data, particularly with the simulated model of AA stacking, further confirming the successful synthesis of TAPB-DHPA and its AA stacking structure.

Figure 2d shows the N_2_ adsorption–desorption performance of TAPB-DHPA. The adsorption–desorption curve exhibits a typical type I isotherm, with a specific surface area of 938 m^2^/g (Table 2). Using the non-local density functional theory (NLDFT) method, the average pore size of the material was calculated to be 2.40 nm. Additionally, the NH₃-TPD test revealed the types and strengths of the acidic sites in TAPB-DHPA, with a total acidity of 1.27 mmol/g. The acidic properties of TAPB-DHPA are closely related to the presence of phenolic hydroxyl groups. In TAPB-DHPA, the p-π conjugation effect between the benzene ring and phenolic hydroxyl group reduces the electron cloud density on the oxygen atom, making the hydroxyl hydrogen more prone to ionization, thereby exhibiting significant acidity.

**Figure 3:**
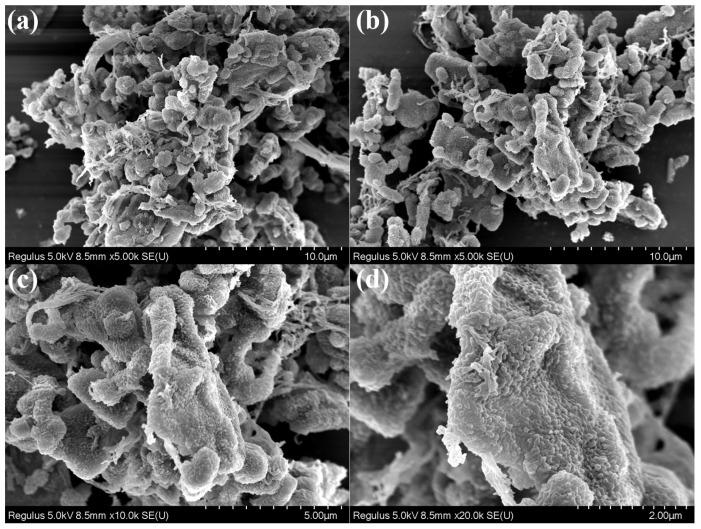
SEM images of TAPB-DHPA at different locations or magnification levels: (**a**,**b**) ×5000; (**c**) ×10,000; (**d**) ×20,000.

Figure 2e shows the thermal stability of TAPB-DHPA. The thermal decomposition temperature of TAPB-DHPA under an N_2_ atmosphere is approximately 400 °C, indicating its excellent thermal stability. This property is closely related to the presence of hydrogen bonds in TAPB-DHPA as the stability of hydrogen bonds effectively enhances the material’s thermal stability. Figure 3 presents the microscopic morphology of TAPB-DHPA. The material exhibits an irregular block-like structure with numerous surface protrusions. The formation of these protrusions is closely related to the Kirkendall effect and Ostwald ripening occurring during the monomer condensation process, which further increases the material’s specific surface area [25].

### 3.3. Catalytic Conversion of XOS to Furfural Using TAPB-DHPA

TAPB-DHPA is a porous material with a high specific surface area and abundant acidic sites, demonstrating significant potential for catalyzing the conversion of XOS to furfural. Therefore, this study optimized the reaction conditions for TAPB-DHPA catalyzed conversion, focusing on key parameters such as solvent system, reaction temperature, catalyst dosage, and reaction time.

**Figure 4:**
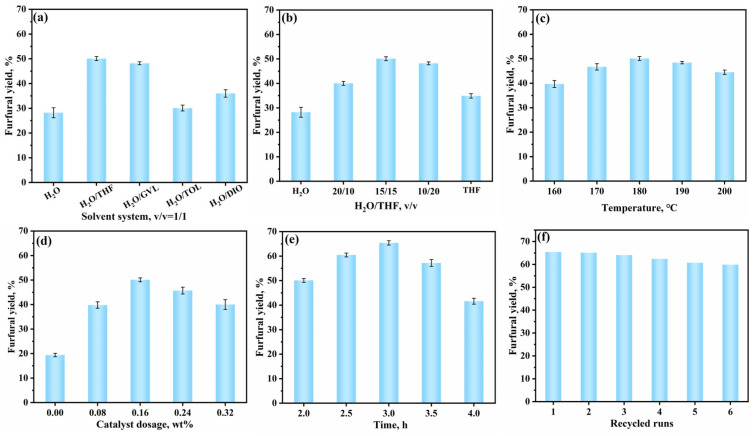
Performance of TAPB-DHPA under different reaction conditions: (**a**) effect of solvent types; (**b**) effect of H_2_O/THF ratio; (**c**) effect of reaction temperature; (**d**) effect of catalyst dosage; (**e**) effect of reaction time; (**f**) reusability of TAPB-DHPA.

Solvent plays a crucial role in the catalytic conversion of XOS to furfural, acting as the medium that directly influences the conversion efficiency [26]. This study first investigated the catalytic performance of TAPB-DHPA in different solvent systems. As shown in Figure 4a, compared to the aqueous phase system (furfural yield of 28.2%), TAPB-DHPA exhibited higher catalytic performance in a biphasic system, indicating that the biphasic system is more favorable for furfural production. This is because the aqueous phase promotes the dissolution and reaction of biomass-derived sugars, while the organic phase effectively suppresses the degradation of furfural. The synergistic effect of both phases maximizes the conversion of biomass-derived sugars to furfural [27]. Specifically, in the H_2_O/THF system, the furfural yield reached its highest level. To further investigate the impact of the water and THF ratio on furfural yield, the results are shown in Figure 4b. As the proportion of THF increased, the yield of furfural first increased and then decreased. When the H_2_O/THF volume ratio was 1:1, the furfural yield peaked at 50.1%. XOS, composed of xylose units linked via β-1,4-glycosidic bonds, exhibit pronounced hydrophilic characteristics due to the dense distribution of hydroxyl groups (-OH) on the molecular surface, making pure water as an ideal reaction medium. However, in monophasic aqueous systems, the aldehyde group of formed furfural establishes strong hydrogen bonds with water molecules, leading to prolonged product residence time and triggering secondary reactions such as resinification polymerization and self-condensation, which significantly reduce selectivity toward the target compound [28]. In contrast, the H_2_O/THF miscible biphasic system enhances furfural yield through synergistic mechanisms [29,30]: (1) the low polarity and hydrogen-bonding donor capacity of THF result in minimal XOS solubility, thereby confining hydrolysis and dehydration reactions to the aqueous phase; (2) the reduced dielectric constant in THF phase facilitates the enrichment of acid catalysts in their undissociated form, stabilizing xylose 1,2-enediol intermediates via hydrogen bonding interactions to selectively promote dehydration; (3) most importantly, the superior polarity matching between THF and furfural enables in situ product extraction into the organic phase, which not only reduces furfural concentration in aqueous phase, thereby suppressing side reactions, but also isolates products from reactants/intermediates through phase separation. This “reaction–extraction” coupling mechanism optimizes the rate matching between hydrolysis and dehydration, effectively preventing xylose over-carbonization. It should be noted that precise control of the THF ratio is essential, as an excessive THF proportion would reduce system acidity and inhibit initial XOS hydrolysis activation.

The effect of temperature on furfural yield is shown in Figure 4c. When the reaction temperature was 160 °C, the furfural yield was 39.7%. As the temperature increased to 180 °C, the yield rose to 50.1%, showing a 10.4% improvement. However, as the temperature continued to rise, the furfural yield began to decrease. The increase in temperature provides the necessary energy for bond cleavage, promoting the reaction. However, when the temperature is too high, side reactions significantly increase, such as the cross-polymerization and self-polymerization of furfural with intermediate products [31]. These side reactions suppress the formation of furfural, leading to a decrease in yield.

Figure 4d shows the effect of catalyst loading on furfural yield. As the amount of TAPB-DHPA increased, the yield of furfural gradually rose, reaching a maximum value when the catalyst loading was 0.16 wt%. However, further increases in catalyst loading led to a decline in yield. This phenomenon can be attributed to the increase in the number of effective active sites in the reaction system, which promotes the conversion of XOS to furfural [32]. However, excessive catalyst amounts may trigger side reactions, even leading to the formation of humins [33], thereby reducing the furfural yield.

Figure 4e shows the effect of reaction time on furfural yield, exhibiting a trend of first increasing and then decreasing. When the reaction time was 3 h, the furfural yield reached its optimum value of 65.4%. Extending the reaction time helps to increase the effective collision probability between molecules, significantly improving the furfural yield. However, further extension of the reaction time may cause the degradation of furfural [34], leading to a decrease in yield.

**Figure 5:**
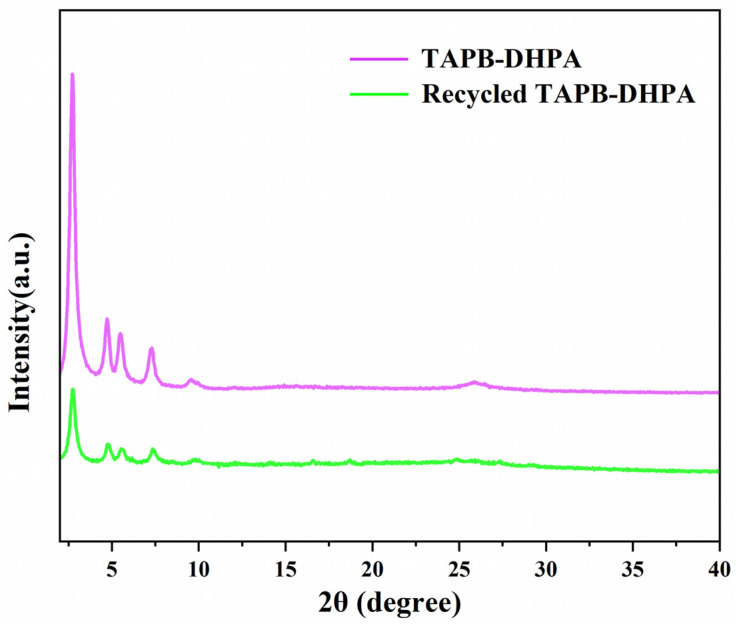
XRD pattern of TAPB-DHPA before and after use.

A significant advantage of TAPB-DHPA as a heterogeneous catalyst is its good reusability. Under optimal reaction conditions, the cycling performance of TAPB-DHPA was evaluated. As shown in Figure 4f, after six cycles, the furfural yield remained above 59.9%, demonstrating the excellent recyclability of TAPB-DHPA. Furthermore, TAPB-DHPA required only simple washing with 1,4-dioxane and n-butanol after use to maintain its original crystal structure (Figure 5), further confirming its excellent stability.

The comparison of the catalytic performance of TAPB-DHPA with other solid acid catalysts is shown in Table 3. Compared to the COF previously synthesized by our research group, TAPB-DHPA can effectively catalyze the conversion of XOS to furfural with a reduced catalyst amount. The furfural yield of TAPB-DHPA is 1.9% higher than that of TP-DAB, primarily due to its larger specific surface area, which provides more exposed active sites. However, its furfural yield is 6.8% lower than that of TAPT-DHPA-COF, a difference that can be attributed to the variations in their acidic functional groups. Compared to other reported solid acid catalysts, TAPB-DHPA exhibits superior catalytic performance. Although the furfural yield of WO_3_/SiO_2_ and PR/C-SO_3_H-Fe is slightly higher than that of TAPB-DHPA, their catalyst amounts are relatively larger. In comparison with Cr^3+^/P-SBA-15 and SCh catalysts, TAPB-DHPA not only shows a higher yield in catalyzing XOS to furfural but also requires a lower catalyst amount. In conclusion, TAPB-DHPA, with its excellent catalytic performance, especially the ability to achieve a high furfural yield at a lower catalyst dose, demonstrates significant potential for application in furfural production.

**Figure 6:**
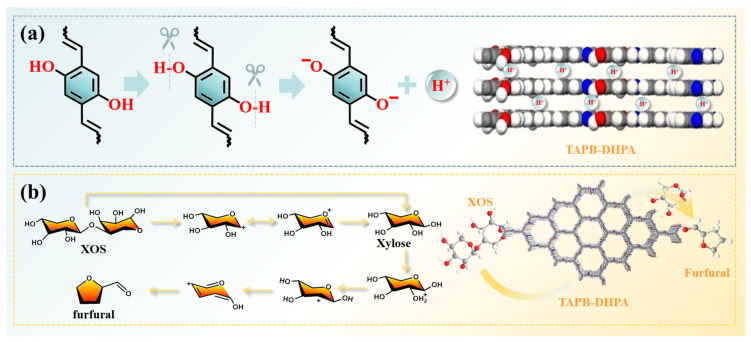
(**a**) Hydrogen ionization-driven acidic catalytic mechanism of TAPB-DHPA. (**b**) The heterogeneous synergistic pathway of TAPB-DHPA for the conversion of XOS to furfural.

In the TAPB-DHPA covalent organic framework, phenolic hydroxyl groups (-OH) derived from the DHPA units undergo proton dissociation facilitated by the p-π conjugation of the aromatic ring (Figure 6a). Specifically, polarization of the O–H bond, induced by the delocalized π-electron system, promoting H⁺ ionization, thereby generating B-acid sites. The resulting phenoxide anion is stabilized through conjugation, which delocalizes the negative charge across the aromatic ring, lowers its energy and significantly reduces the pKa value. Additionally, residual aldehyde groups (-CHO) within the COF layers form intramolecular hydrogen bonds with adjacent phenolic hydroxyls, further activating the O-H bonds and facilitating proton release. These B-acid sites, synergistically stabilized by both conjugation effects and hydrogen-bonding networks, enable efficient tandem hydrolysis–dehydration catalysis for xylan conversion. The high local concentration of protons in the nanoconfined spaces markedly accelerates the reaction rate, offering a novel heterogeneous catalytic strategy for the green synthesis of furfural, a key biomass-derived platform chemical.

The possible reaction mechanism of TAPB-DHPA catalyzing the conversion of XOS to furfural is as follows. TAPB-DHPA, as a solid acid catalyst containing B-acid, catalyzes the production of furfural primarily through hydrolysis and dehydration reactions [39]. The hydrolysis reaction refers to the cleavage of the β-1,4-glycosidic bond in xylobiose under the catalysis of B-acid, resulting in the formation of xylose [27,40]. The specific reaction mechanism is shown in Figure 6b. Under the catalytic action of B-acid, the oxygen atom in the β-1,4-glycosidic bond of xylobiose is first protonated, followed by the cleavage of the C-O bond, forming xylose and a carbon cation intermediate. The carbon cation then undergoes electron transfer to form a double bond, and ultimately, under the influence of B-acid, a water molecule adds to the double bond, resulting in the formation of xylose. After hydrolysis, xylose undergoes further dehydration under B-acid catalysis to be converted into furfural [41,42]. The possible dehydration steps are shown in Figure 6b. Under the catalysis of B-acid, the hydroxyl oxygen of xylose is protonated and dehydrated, forming a carbon cation. Subsequently, the C-O bond breaks, and the carbon cation forms a C=C double bond with the adjacent carbon atom, while oxygen forms a C=O double bond with another carbon atom and releases a proton. The proton continues to attack the hydroxyl oxygen, generating two water molecules: a C=C bond and a new carbon cation. Finally, through an elimination reaction, the molecule cyclizes to form furfural. In summary, TAPB-DHPA catalyzes the hydrolysis and dehydration reactions through its B-acid sites, converting xylobiose into xylose, which is subsequently catalyzed to further transform into furfural, providing an efficient conversion pathway for this study.

## 4. Conclusions

In this study, a hydroxyl-functionalized COF, TAPB-DHPA, was successfully synthesized via solvothermal method and innovatively applied to catalyze the conversion of XOS in pulp pre-hydrolysis liquor into furfural. The results demonstrate that TAPB-DHPA possesses a high specific surface area, porous structure, excellent thermal stability, and abundant acidic sites, making it an ideal solid acid catalyst. In a H_2_O/THF (*v*/*v* = 1/1) mixed solvent system, the TAPB-DHPA catalyst was employed for the efficient conversion of XOS to furfural. Under optimized reaction conditions (catalyst loading: 0.16 wt%; temperature: 180 °C, time: 3 h), the furfural yield reached 65.4%, with the catalyst maintaining over 59.9% of its catalytic activity after six consecutive cycles. The high efficiency of this catalytic system is attributed to the acidic microenvironment provided by the phenolic hydroxyl groups, which effectively promote the hydrolysis–dehydration cascade reactions of XOS. Additionally, the presence of THF modulates solvent polarity and facilitates in situ extraction of generated furfural, significantly reducing its concentration in the polar aqueous phase and thereby suppressing side reactions such as resinification. This study not only offers an efficient catalytic system for green furfural production but also introduces a novel pathway for the high-value utilization of waste biomass from the pulp and paper industry, demonstrating considerable industrial application potential.

## Figures and Tables

**Table 1:** Basic properties of XOS.

Sample	Moisture Content (%)	Purity (%)	Mw (g/mol)	Mn (g/mol)	PDI
XOS	0.44	96.7	306	286	1.07

**Table 2:** BET, pore sizes, total acidity (TA), and thermal degradation temperature (T) of TAPB-DHPA.

Synthesized COF	BET (m^2^/g)	Pore Sizes (nm)	TA (mmol/g)	T (°C)
TAPB-DHPA	938	2.40	1.27	400

**Table 3:** Comparison of the catalytic performance of TAPB-DHPA with other solid acid catalysts.

Catalyst	Catalyst Dosage	Reaction	Yield	Ref.
TAPB-DHPA	0.16 wt%	180 °C, 180 min	65.4%	This work
TP-DAB	0.33 wt%	160 °C, 180 min	63.5%	[14]
TAPT-DHPA-COF	0.33 wt%	200 °C, 90 min	72.2%	[13]
WO_3_/SiO_2_	10 wt%	170 °C, 10 h	71.0%	[35]
PR/C-SO_3_H-Fe	0.71 wt%	160 °C, 180 min	79.0%	[36]
Cr^3+^/P-SBA-15	40 wt%	180 °C, 120 min	58.0%	[37]
SCh	0.50 wt%	190 °C, 80 min	42.0%	[38]

## Data Availability

Data supporting reported that the results can be obtained from the authors on request.

## References

[1] Liu S.Q., Meng Y., Li H., Yang S. (2021). Hierarchical Porous MIL-101(Cr) Solid Acid-Catalyzed Production of Value-Added Acetals from Biomass-Derived Furfural. Polymers.

[2] Puke M., Godina D., Brazdausks P. (2024). Catalyzed Hydrothermal Pretreatment of Oat Husks for Integrated Production of Furfural and Lignocellulosic Residue. Polymers.

[3] Cunha A.E.P., Simoes R.M.S. (2023). Dissolving Kraft Pulp Production and Xylooligosaccharide Coproduction: Effect of Pre-Hydrolysis Conditions. Acs Omega.

[4] Huijgen W.J.J., Smit A.T., de Wild P.J., den Uil H. (2012). Fractionation of wheat straw by prehydrolysis, organosolv delignification and enzymatic hydrolysis for production of sugars and lignin. Bioresour. Technol..

[5] Lou R., Zhang X. (2022). Evaluation of pretreatment effect on lignin extraction from wheat straw by deep eutectic solvent. Bioresour. Technol..

[6] Yang L., Shao L.S., Wu Z.P., Zhan P., Zhang L. (2023). Design and Synthesis of Porous Organic Polymers: Promising Catalysts for Lignocellulose Conversion to 5-Hydroxymethylfurfural and Derivates. Polymers.

[7] Chai Y.D., Pang Y.L., Lim S., Chong W.C., Lai C.W., Abdullah A.Z. (2022). Recent Progress on Tailoring the Biomass-Derived Cellulose Hybrid Composite Photocatalysts. Polymers.

[8] Gan P., Zhang K., Yang G.H., Li J.Z., Zhao Y., Chen J.C. (2024). Catalytic Production and Upgrading of Furfural: A Platform Compound. Int. J. Mol. Sci..

[9] Deng W.P., Feng Y.C., Fu J., Guo H.W., Guo Y., Han B.X., Jiang Z.C., Kong L.Z., Li C.Z., Liu H.C. (2023). Catalytic conversion of lignocellulosic biomass into chemicals and fuels. Green Energy Environ..

[10] Ke K., Ji H.R., Shen X.N., Kong F.O., Li B. (2021). Pressure Reduction Enhancing the Production of 5-Hydroxymethylfurfural from Glucose in Aqueous Phase Catalysis System. Polymers.

[11] Peng Y.W., Hu Z.G., Gao Y.J., Yuan D.Q., Kang Z.X., Qian Y.H., Yan N., Zhao D. (2015). Synthesis of a Sulfonated Two-Dimensional Covalent Organic Framework as an Efficient Solid Acid Catalyst for Biobased Chemical Conversion. ChemSusChem.

[12] Du Y.R., Xu B.H., Pan J.S., Wu Y.W., Peng X.M., Wang Y.F., Zhang S.J. (2019). Confinement of Bronsted acidic ionic liquids into covalent organic frameworks as a catalyst for dehydrative formation of isosorbide from sorbitol. Green Chem..

[13] Gan P., Zhang K., Li Z.H., Zhang C.X., Yang G.H., Zhang L., Wang B.B., Chen J.C. (2024). One-pot furfural production from sustainable biomass-derived sugars using a functionalized covalent organic framework as a heterogeneous catalyst. Green Chem..

[14] Ju Y., Zhang K., Gan P., Guo B.Z., Xia H.M., Zhang L.L., Wang B.B., Zhang L., Chen J.C. (2025). Functionalized covalent organic frameworks for catalytic conversion of biomass-derived xylan to furfural. Int. J. Biol. Macromol..

[15] Pang J.X., Zhang Y., Tong X.Y., Zhong Y.G., Kong F.J., Li D., Liu X.F., Qiao Y.J. (2023). Recent Developments in Molecular Characterization, Bioactivity, and Application of Arabinoxylans from Different Sources. Polymers.

[16] Chen Z.W., Hu T.Q., Jang H.F., Grant E. (2015). Modification of xylan in alkaline treated bleached hardwood kraft pulps as classified by attenuated total-internal-reflection (AIR) FTIR spectroscopy. Carbohydr. Polym..

[17] Åkerholm M., Hinterstoisser B., Salmén L. (2004). Characterization of the crystalline structure of cellulose using static and dynamic FT-IR spectroscopy. Carbohydr. Res..

[18] Chen H., Ferrari C., Angiuli M., Yao J., Raspi C., Bramanti E. (2010). Qualitative and quantitative analysis of wood samples by Fourier transform infrared spectroscopy and multivariate analysis. Carbohydr. Polym..

[19] Palaniappan A., Balasubramaniam V.G., Antony U. (2017). Prebiotic potential of xylooligosaccharides derived from finger millet seed coat. Food Biotechnol..

[20] Yang D., Zhong L.X., Yuan T.Q., Peng X.W., Sun R.C. (2013). Studies on the structural characterization of lignin, hemicelluloses and cellulose fractionated by ionic liquid followed by alkaline extraction from bamboo. Ind. Crop. Prod..

[21] Lu F.F., Wu M.Q., Lin C.C., Lin X.C., Xie Z.H. (2022). Efficient and selective solid-phase microextraction of polychlorinated biphenyls by using a three-dimensional covalent organic framework as functional coating. J. Chromatogr. A.

[22] Wang H.Z., Yang C., Chen F.S., Zheng G.F., Han Q. (2022). A Crystalline Partially Fluorinated Triazine Covalent Organic Framework for Efficient Photosynthesis of Hydrogen Peroxide. Angew. Chem. Int. Ed..

[23] Kuang L.J., Wang S.Q., Wan H.F., Chen L.L., Wang L., Song Y.H. (2023). Designing Fluorescent Covalent Organic Frameworks by Controlling Layer Spacing, Size of Aromatic Linker and Side Chains for Detection of Nitrofurazone. Adv. Opt. Mater..

[24] Hu H., Tao Y.L., Wang D., Li C.L., Jiang Q.C., Shi Y.X., Wang J., Qin J.P., Zhou S.J., Kong Y. (2023). Rational modification of hydroxy-functionalized covalent organic frameworks for enhanced photocatalytic hydrogen peroxide evolution. J. Colloid Interface Sci..

[25] Kandambeth S., Venkatesh V., Shinde D.B., Kumari S., Halder A., Verma S., Banerjee R. (2015). Self-templated chemically stable hollow spherical covalent organic framework. Nat. Commun..

[26] Shen G.F., Andrioletti B., Queneau Y. (2020). Furfural and 5-(hydroxymethyl)furfural: Two pivotal intermediates for bio-based chemistry. Curr. Opin. Green Sustain. Chem..

[27] Zhao Y., Lu K.F., Xu H., Zhu L.J., Wang S.R. (2021). A critical review of recent advances in the production of furfural and 5-hydroxymethylfurfural from lignocellulosic biomass through homogeneous catalytic hydrothermal conversion. Renew. Sust. Energ. Rev..

[28] Lin Q.X., Zhan Q.W., Li R., Liao S.W., Ren J.L., Peng F., Li L.B. (2021). Solvent effect on xylose-to-furfural reaction in biphasic systems: Combined experiments with theoretical calculations. Green Chem..

[29] Yu I.K.M., Tsang D.C.W., Chen S.S., Wang L., Hunt A.J., Sherwood J., Vigier K.D., Jerome F., Ok Y.S., Poon C.S. (2017). Polar aprotic solvent-water mixture as the medium for catalytic production of hydroxymethylfurfural (HMF) from bread waste. Bioresour. Technol..

[30] Weingarten R., Cho J., Conner W.C., Huber G.W. (2010). Kinetics of furfural production by dehydration of xylose in a biphasic reactor with microwave heating. Green Chem..

[31] Pang S.H., Medlin J.W. (2011). Adsorption and Reaction of Furfural and Furfuryl Alcohol on Pd(111): Unique Reaction Pathways for Multifunctional Reagents. ACS Catal..

[32] Xu Z.P., Li W.Z., Du Z.J., Wu H., Jameel H., Chang H.M., Ma L.L. (2015). Conversion of corn stalk into furfural using a novel heterogeneous strong acid catalyst in γ-valerolactone. Bioresour. Technol..

[33] Yang R., Zhang H.T., Li X.D., Ye X.G., Liu L. (2024). When Furfural Meets Piancatelli Rearrangement. ACS Sustain. Chem. Eng..

[34] Hu B., Zhang W.M., Wang X.G., Zhang B., Liu J., Lu Q. (2023). The formation mechanism of furfural in xylan pyrolysis: A machine learning study based on neural network potential. Fuel Process. Technol..

[35] Bhaumik P., Dhepe P.L. (2017). From Lignocellulosic Biomass to Furfural: Insight into the Active Species of a Silica-Supported Tungsten Oxide Catalyst. Chemcatchem.

[36] Gai X.T., Ding W., He J., Guo J., Song K. (2025). Furfural production from xylan using a Pueraria Residues carbon-based solid-acid catalyst. J. Sci. Food Agric..

[37] Xu S., Pan D., Wu Y., Fan J., Wu N., Gao L., Li W., Xiao G. (2019). Catalytic Conversion of Xylose and Xylan into Furfural Over Cr3+/P-SBA-15 Catalyst Derived from Spent Adsorbent. Ind. Eng. Chem. Res..

[38] Wang Y., Delbecq F., Kwapinski W., Len C. (2017). Application of sulfonated carbon-based catalyst for the furfural production from d-xylose and xylan in a microwave-assisted biphasic reaction. Mol. Catal..

[39] Ye L., Han Y.W., Wang X.T., Lu X.B., Qi X.H., Yu H.B. (2021). Recent progress in furfural production from hemicellulose and its derivatives: Conversion mechanism, catalytic system, solvent selection. Mol. Catal..

[40] Fan X.Y., Ren M.N., Zhou C.S., Kong F.G., Hua C.H., Fakayode O.A., Okonkwo C.E., Li H.X., Liang J.K., Wang X. (2024). Total utilization of lignocellulosic biomass with xylooligosaccharides production priority: A review. Biomass Bioenergy.

[41] Xu C., Paone E., Rodriguez-Padron D., Luque R., Mauriello F. (2020). Recent catalytic routes for the preparation and the upgrading of biomass derived furfural and 5-hydroxymethylfurfural. Chem. Soc. Rev..

[42] Mamman A.S., Lee J.M., Kim Y.C., Hwang I.T., Park N.J., Hwang Y.K., Chang J.S., Hwang J.S. (2008). Furfural: Hemicellulose/xylosederived biochemical. Biofuels Bioprod. Biorefining.

